# Can Agricultural Cooperatives Promote Chinese Farmers’ Adoption of Green Technologies?

**DOI:** 10.3390/ijerph20054051

**Published:** 2023-02-24

**Authors:** Chong Dong, Hainan Wang, Wenjin Long, Jiujie Ma, Yi Cui

**Affiliations:** 1 Institute of Rural Development, Chinese Academy of Social Sciences, Beijing 100732, China; 2 National Agricultural Exhibition Center, Beijing 100125, China; 3 School of Economics and Management, China Agricultural University, Beijing 100083, China; 4 School of Agricultural Economics and Rural Development, Renmin University of China, Beijing 100872, China; 5 School of Economics, Beijing Technology and Business University, Beijing 100048, China

**Keywords:** green technology, technology adoption behavior, agricultural cooperatives, water-saving irrigation technology, commercial organic fertilizer

## Abstract

Green technologies are important for achieving green and high-quality agricultural development. The Chinese government has issued various policies to explicitly encourage the adoption of green technologies. However, incentives for Chinese farmers to adopt green technologies remain insufficient. This study examines whether participation in agricultural cooperatives can help break the barriers to Chinese farmers’ adoption of green technologies. It also examines the potential mechanisms by which cooperatives can mitigate the lack of incentives for farmers to adopt agricultural green technologies. Using data from a study on farmers in four Chinese provinces, we found that cooperative participation significantly increases farmers’ adoption behavior for both green technologies with effective market incentives (e.g., commercial organic fertilizer technologies) and those without such incentives (e.g., water-saving irrigation technologies).

## 1. Introduction

Since the United Nations adopted the 2030 Agenda for Sustainable Development [1], on 25 September 2015, green development has become a global priority. Simultaneously, the green development of agriculture has also received increasing attention from governments worldwide, while the construction of a technology system to support the green development of agriculture and the promotion of green technologies in agriculture has become an important topic of discussion in policymaking.

Although the Chinese government has attached great importance to green and high-quality agricultural development in recent years, the reliance on high input and the consumption of resources to achieve high agricultural output has not fundamentally changed, while smallholder farmers remain insufficiently motivated to adopt green technologies. According to data published by China’s National Bureau of Statistics, the amount of fertilizer used for agriculture in China reached 198 kg/ha in 2019, which is much higher than that in the United States (72 kg/ha), Canada (66 kg/ha), and Australia (43 kg/ha) [2]. Further, the intensity of fertilizer application in China exceeds internationally recognized safety limits. 

Meanwhile, as a result of the widespread use of inefficient irrigation methods, almost all available irrigation water, including surface water and groundwater, is used to meet irrigation needs as much as possible. The problem of the low utilization of agricultural production inputs in China is also prominent. The Ministry of Water Resources of the People’s Republic of China estimated that the effective utilization rate of irrigation water in China’s farmlands would be only 54.2% by 2020 [3]. Excessive input of chemicals and the inefficient use of natural resources lead to ecological degradation and overconsumption of natural resources, as well as various quality and safety risks for agricultural products.

The adoption of green technologies in agriculture can avoid the misuse and pollution of public resources and help ensure public food safety. For example, the adoption of water-saving irrigation technologies can save water resources, while the adoption of organic fertilizers can improve land quality and contribute to the safety and quality of agricultural products and consumer health [4,5]. Since the property rights of public resources (e.g., irrigation water) and public goods (e.g., public food security) are difficult to clearly define, farmers have incentives to use them competitively or act as “free riders” [6]. This can lead to the overuse of public resources and the undersupply or damage of public goods. 

Theoretically, the adoption of technology by farmers is conditional on the marginal private cost being equal to the marginal private benefit. However, due to the positive externalities of green technologies, the marginal social benefits of adopting green technologies are higher than their marginal private benefits. Therefore, farmers do not have natural incentives to adopt green technologies. In addressing this issue, the institutional arrangement of agriculture cooperatives has some advantages.

In China, where there is a large number of smallholder farmers, cooperatives can be effective in promoting the adoption of green technologies by increasing the efficiency of internalizing the external benefits of green farming technology adoption. Cooperative members have a common economic purpose and therefore have an endogenous incentive to promote standardized agricultural production. Cooperatives can also make effective use of multiple institutional arrangements such as repeated games, reputation mechanisms, incentives, and traceability mechanisms to ensure the quality and safety of agricultural products, and reduce opportunistic behavior of members through appropriate incentive payment strategies, thereby providing incentives for members to follow higher green production standards and receive a more reasonable premium for quality. At the same time, cooperatives can promote investment in and use of dedicated assets, reducing the cost of adopting green technologies for farmers and mitigating the diseconomies of adopting dedicated assets. In addition, cooperatives can promote green technology diffusion through technical training, breaking the knowledge barrier for farmers to adopt green farming technologies. 

The negative externalities of resource misuse can be eliminated through market-based transactions or negotiations in the private sector, thereby achieving optimal resource allocation. When a specific market exists to realize the value of green agricultural technology adoption, the social benefits generated by the adoption of certain green technologies can be transformed into real benefits for adopters through trading behaviors. This enables an effective market incentive to adopt green technology. For example, if there is a market for high-value-added quality agricultural products, the private costs incurred by farmers in adopting green agricultural technologies such as green organic fertilizers and bio-pesticides for the production of quality agricultural products can be compensated by the higher market transaction value, and the expectation of higher private gains for farmers will provide further incentives for farmers to adopt these green agricultural technologies.

However, in reality, due to information asymmetry, farmers are often unable to connect with the premium realization market effectively; thus, the market incentive cannot function effectively, thereby resulting in a lack of incentives for farmers to adopt green technologies or the prevalence of free-riding. 

In this case, cooperatives can establish and optimize economic incentives through the advantages of property rights and effective governance. On the one hand, compared with smallholder farmers, cooperatives can more easily connect with the premium market for green technology adoption, thus increasing the private marginal returns of farmers and enhancing their economic incentives to adopt green technologies. On the other hand, cooperatives’ common property rights system can reduce the principal-agent cost caused by the mismatch between residual income claims and residual control, as well as create a good incentive and constraint mechanism to inhibit cooperative parties’ opportunistic tendencies and free-riding behavior [7].

When there is no market for realizing the value of green technology adoption or the development of such a market is seriously delayed, the social benefits of adopting such technologies cannot be transformed into real benefits, as there is no effective market incentive. In this case, cooperatives’ common property rights system can alleviate the uneconomical problem of adopting relevant dedicated assets and lower the cost and threshold for farmers to adopt green technologies through socialized services, thus establishing an economic incentive for farmers’ adoption behavior. 

Furthermore, the impact of noneconomic factors such as information friction is more important because there is no effective market incentive [8]. Some studies have shown that the adoption of green technologies by producers is usually lower than that expected from policy interventions or economic inducements alone [9]. On the one hand, values and pro-environmental intentions are important factors influencing farmers’ adoption of green technologies. When farmers’ awareness of environmental protection is low, neither government policy guidance nor market incentives may be sufficient to promote green technology adoption. On the other hand, the uncertainty of the rewards of adopting green technologies is high, while incomplete or imperfect information will increase the uncertainty of farmers’ benefits, which further restricts the diffusion of green agricultural technologies [10]. Cooperatives’ behavioral nudges to mitigate information frictions include educating farmers [11,12] to reduce the uncertainty in farmers’ perceptions of technology [13], as well as shaping social norms at the community level to promote their adoption of green technologies [14].

Some studies have found that cooperatives have advantages in encouraging farmers to adopt green technologies. Cooperatives help members control chemical inputs and adopt various conservation and pollution prevention measures [15]. Cooperative members are more likely to adopt green production technologies in terms of land conservation, fertilizer inputs, etc. [16]. However, some studies have indicated that cooperatives’ impact on farmers’ adoption of green technology is insignificant or uncertain. A study on Ugandan farmers showed that participation in cooperatives did not significantly contribute to the adoption of organic fertilizers due to the imperfect development of cooperatives and the fundamental differences between members and non-members, which influence their participation in cooperative decision-making [17]. Additionally, cooperatives improve market competitiveness and bargaining power through organizations, thus increasing the producer prices of agricultural products. Farmers may sacrifice the ecological and social benefits of agriculture in pursuit of higher yields and incomes [18].

This study explores whether participation in cooperatives can promote farmers’ adoption of green technologies and what mechanisms cooperatives can use to alleviate the lack of incentives for farmers to adopt different types of green technologies. Based on data from a 2018 study on 411 farmers in four Chinese provinces, the present study finds that farmers’ participation in cooperatives significantly enhances their adoption behavior for both green technologies for which effective market incentives exist (e.g., commercial organic fertilizer technologies) and those for which such incentives do not exist (e.g., water-saving irrigation technologies).

The main contribution of this study is that it discusses the influence of cooperatives on farmers’ adoption of green technology and its mechanism, thereby enriching the theoretical knowledge of this relationship and its mechanism. Furthermore, it may provide valuable insights for policymakers in developing countries aiming to improve green technology promotion policies and rationalize the role of cooperatives, which could help alleviate agricultural surface pollution, protect and utilize agricultural resources, restore agricultural ecology, as well as improve agricultural product quality and safety and public health.

## 2. Data and Methods

### 2.1. Data

The data used in this study was derived from research carried out in 2018 on micro-level farm households. A stratified sampling method was used to select the study participants. To make the sample more representative, we selected Shandong Province (East China), Hebei Province (North China), Sichuan Province (Southwest China), and Gansu Province (Northwest China), considering the differences in the structure of the farming industry and cooperatives’ development in each region. The sampling site in Shandong belongs to the plain area, with a continental climate in the warm-temperate monsoon zone, dry in spring and autumn with little rain, hot and rainy in summer, with a well-developed cash crop farming industry and a prosperous agricultural market; the sampling site in Hebei belongs to the hilly area, with a continental climate in the warm-temperate semi-arid monsoon zone, with abundant irrigation water resources and a relatively well-developed grain crop farming industry; the sampling site in Sichuan belongs to the mountainous area, with a humid monsoon climate in the central subtropical zone. The climate is mild, with warm winters, long summers, and abundant rainfall, and crops can be grown in all four seasons. The sampling area in Gansu belongs to the plateau region, which belongs to the transition zone from a temperate semi-humid to an alpine humid climate, with high altitude, relatively appropriate rainfall, low temperature, abundant groundwater resources, and relatively well-developed cultivation of cold-loving food crops and Chinese herbal medicine.

These four provinces exhibit differences in their levels of economic development, the structure of their respective agricultural industries, and their cooperatives’ development level. According to the National Farmers’ Cooperative Demonstration Society Development Index Report (2018) [19], released by the Ministry of Agriculture and Rural Affairs, Hebei and Sichuan occupy six seats, Gansu five seats, and Shandong three seats in the list of the top 100 national farmers’ cooperative demonstration societies. 

On this basis, we included in the sample one county (county-level city) from each province. The inclusion criteria were the following: (1) counties that were mainly engaged in plantation production and (2) whose percentage of the added value of the agricultural industry in their regional Gross Domestic Product (GDP) exceeded the national average (7.9%), according to 2017 data [20]. Consequently, Shouguang City in Shandong, Shunping County in Hebei, Yilong County in Sichuan, and Min County in Gansu were chosen. 

In each county, the townships were divided into economically developed and economically less developed groups, according to the per capita GDP, while one township was randomly selected in each group. Further, in each township, the administrative villages were divided into high-income, middle and high-income, and middle and low-income, according to the per capita net income level, while one village was randomly selected in each group. Moreover, in each village, 14 farming households were randomly selected according to the villagers’ roster according to the equidistant sampling method. The survey took the form of one-on-one interviews and 411 valid questionnaires were collected.

According to the main data bulletin of the third National Agricultural Census, by the end of 2017, the number of agricultural cooperatives registered in the industrial and commercial sector had reached 1.933 million, with more than 100 million farmer participants, accounting for approximately 46.8% of all farmers nationwide, while small households in China accounted for 98.1% of the total number of agricultural business households. Therefore, the participation rate of small households in cooperatives at the national level was calculated to be approximately 46.0%. In the data used in this study, farmers participating in cooperatives accounted for 50.9% of the sample farmers, which is close to national-level data. 

The present study collected the production information of farmers in the last full crop growing season, so the interference of the crop growing season on the estimation results was controlled after controlling for the crop type.

In summary, the sample selection in this study was reasonable and the sample data had good representativeness.

### 2.2. Methodology

#### 2.2.1. Identification Strategy

In estimating the effect of farmers’ participation in cooperatives on their adoption of green technologies, the following model was constructed in this paper.

*y_i_* = *α*_0_ + *α*_1_ × *Membership_i_* + *α*_2_ × *X*_i_ + *ε_i_*. (1)

In Equation (1), *y_i_* denotes the behavioral performance of a farmer in adopting a given green technology; if the farmer adopts said technology, then *y_i_* = 1, otherwise *y_i_* = 0. The core explanatory variable *Membership_i_* is a dummy variable; if a farmer participates in a cooperative, then *Membership_i_* = 1, otherwise *Membership_i_* = 0. Moreover, *X_i_* is a set of control variables reflecting personal characteristics, family characteristics, and production and operation characteristics. Additionally, this study controlled for province-fixed effects. Considering that the explanatory variable is a dummy variable, the ordinary least squares (OLS), Probit, and endogenous switching Probit (ESP) models were used for estimation in this study.

There are three difficulties in identifying the causal relationship between farmers’ participation in cooperatives and their adoption of green technology. The first is the self-selection problem. Farmers’ participation or non-participation in cooperatives is not randomly assigned but the result of farmers’ self-selection, so there may be differences in the initial conditions of the treatment and control groups. 

Second, there is the problem of missing data. To estimate the effect of farmers’ participation in cooperatives on their adoption of green technologies, it would be necessary to compare the adoption behavior of farmers who participate in cooperatives and their adoption behavior in a non-participation scenario. However, it is not possible to observe farmers’ technology adoption behavior in both participating and non-participating conditions, which could lead us to a missing data problem that may lead to sample selection bias if not handled properly.

Third, we consider the problem of omitted variables. The biggest endogeneity concern in this paper is that farmers’ participation in a cooperative and their green technology adoption may be jointly influenced by omitted variables, which may include both observable and unobservable variables.

In addressing these issues, both OLS and Probit estimation methods have shortcomings. The endogeneity between cooperative participation and the adoption of green technologies could not be resolved, while farmers’ cooperative participation was treated as an exogenous variable. Furthermore, the OLS model mainly focuses on the dependent variable as a continuous variable, so there are inevitably large estimation errors. Considering the existing literature and the data in this study, as the adoption of green technology is a discrete variable, this study adopts the ESP model [21]. The ESP consists of two models: one for the selection equation (whether to participate in the cooperative) and one for the outcome equation (farmers’ adoption of green technologies). The basic principle is to add the Mills’ ratio calculated in the first stage (selection equation) to the outcome equation by using the full information maximum likelihood method, fit the outcome equation of the treatment group and the control group separately, and then calculate the treatment effect of the treatment variable (membership in cooperatives) on the explanatory variable (farmers’ adoption of green technology) by constructing a “counterfactual” framework. The effect of the treatment variable (cooperative membership) on the explanatory variable (farmers’ green technology adoption) was calculated by constructing a counterfactual framework.

#### 2.2.2. Model Setting and Estimation Process

In discussing the impact of farmers’ participation in cooperatives on their adoption of a specific green technology, this study uses a random utility framework to analyze farmers’ decisions [18]. In this framework, the actual effect of farmers’ participation in cooperatives is unknown. However, if the effect of a farmer’s participation in a cooperative, *U_i_^m^*, is greater than the effect of not participating in a cooperative, *U_i_^n^*, the farmer will choose to participate in the cooperative. Assuming that I^*^ = U_i_^m^ − U_i_^n^, I^*^ can be expressed as a latent variable, the observed variable *Z_i_* in the model is a function of Equation (2) as follows.

I^*^ = *γZ_i_* + *μ_i_* (2)

I^*^ is the latent variable for whether the farmer participates in the cooperative, when I^*^ > 0, *I_i_ =* 1, the farmer participates in the cooperative; when I^*^ < 0*, I_i_ =* 0*,* the farmer does not participate in the cooperative. *Z_i_* is a vector of variables influencing the farmer’s choice of whether to participate in the cooperative. *γ* is the corresponding coefficient, and*μ_i_* is a random error term with expectation 0.

The outcome equations for farmers in different states are:

*y*_1i_^*^ = *β*_1_*X*_1i_ + *ε*_1*i*_, *y*_1i_^*^ = *I*(*y*_1i_^*^ > 0) (3)

*y*_0i_^*^ = *β*_0_*X*_0*i*_ + *ε*_0*i*_, *y*_0i_^*^ = *I*(*y*_0i_^*^ > 0) (4)

where *y*_1i_^*^ and *y*_0i_^*^ are latent variables (propensity to adopt green technology) that determine the outcome variable (whether or not to adopt green technologies). Further, *X*_1*i*_ and *X*_0*i*_ are vectors of weakly exogenous variables, assuming that *X*_1*i*_ and *X*_0*i*_ contain the same variables. Additionally, *β*_1_ and *β*_0_ are the coefficient vectors, while *ε*_1*i*_ and *ε*_0*i*_ are the error terms.

Assuming that *μ_i_*, *ε*_1*i*_, and *ε*_0*i*_ are jointly normally distributed with a mean of zero, the matrix of correlation coefficients is as follows:

(5) $$\Omega =\left(\begin{array}{ccc} 1 & \rho_{0} & \rho_{1}\\ & 1 & \rho_{10}\\ & & 1 \end{array}\right)$$

where *ρ*_0_ is the correlation coefficient between *ε*_0_ and*μ_i_*, *ρ*_1_ is the correlation coefficient between *ε*_1_ and *μ_i_*, and *ρ*_10_ is the correlation coefficient between *ε*_0_ and *ε*_1_. Since *y*_0*i*_ and *y*_1*i*_ could not be observed simultaneously and the joint distribution of (*ε*_0*i*_*, ε*_1*i*_) could not be confirmed, it was not possible to estimate *ρ*_10_, assuming *ρ*_10_ *=* 1.

Based on the selection and outcome equations, the probability that a farmer will adopt a given green technology, conditional on their participation in the cooperative, can be calculated as:

Pr(*y* = 1|*T* = 1, *X* = *x*) = *ϕ*(*zγ*, *β*_1_*X*_1_, *ρ*)/*F*(*zγ*) (6)

Similarly, the probability that a farmer adopts a given green technology, conditional on not participating in a cooperative, is:

Pr(*y* = 1|*T* = 0, *X* = *x*) = *ϕ*(−*zγ*, *β*_0_*X*_0_, −*ρ*)/*F*(−*zγ*) (7)

Considering the possible endogeneity between farmers’ choice to participate in a cooperative and the adoption of green technologies, this study constructed a log-likelihood function and used the method of great likelihood to obtain consistent estimates, finding the treatment effects of farmers’ participation in cooperatives on the adoption of green technology based on the estimated coefficient vectors.

The (individual) treatment effect of farmers’ participation in a cooperative on their adoption of green technology in the treatment group sample (*T* = 1) is *TT*.

*TT*(*x*) = Pr(*y*_1_ = 1|*T* = 1, *X* = *x*) − Pr(*y*_0_ = 1|*T* = 0, *X* = *x*) = [*φ*(*zγ*, *β*_1_*X*_1_, *ρ*_1_) − *φ*(*zγ*, *β*_0_*X*_0_, *ρ*_0_)]/*F*(*zγ*) (8)

where *F* is the cumulative distribution function of the univariate normal distribution.

The (individual) treatment effect of farmers’ participation in cooperatives on their adoption of green technology for the control group sample (*T* = 0) is *TU*.

*TU*(*x*) = Pr(*y*_1_ = 1|*T* = 0, *X* = *x*) − Pr(*y*_0_ = 1|*T* = 0, *X* = *x*) = [*φ*(−*zγ, β*_1_*X*_1_*,* −*ρ*_1_) −*φ*(−*zγ, β*_0_*X*_0_*,* −*ρ*_0_)]*/F(−zγ*) (9)

The (individual) treatment effect of farmers’ participation in cooperatives on their adoption of green technology in the full sample was:

*TE*(*x*) = Pr(*T* = 1, *X* = *x*) − Pr(*T* = 0, *X* = *x*) = *F*(β_1_*X*_1_) − *F*(*β*_0_*X*_0_) (10)

The average treatment effects for the different groups (*ATT*, *ATE*, and *ATU*) were calculated using the three equations described above.

(11) $$ATT=\frac{1}{N_{T}}\sum_{i=1}^{N_{T}}TT(x_{i})$$

where *ATT* is the treatment effect of farmers’ cooperative participation in the adoption of green technology in the treatment group, while *N_T_* is the number of farmers in the treatment group.

(12) $$ATU=\frac{1}{N_{U}}\sum_{i=1}^{N_{U}}TU(x_{i})$$

where *ATU* is the treatment effect of farmers in the control group participating in the cooperative on the adoption of green technology, while *N_U_* is the number of farmers in the control group.

(13) $$ATE=\frac{1}{N_{E}}\sum_{i=1}^{N_{E}}TE(x_{i})$$

where *ATE* is the treatment effect of all farmers’ participation in the cooperative on the adoption of green technology, while *N* is the number of farmers in the full sample.

### 2.3. Variable Description

#### 2.3.1. Outcome Variables

The adoption of green technologies by farmers implies higher technology conversion costs and use costs, as well as a possible loss of yield. However, for green agricultural technologies for which effective market incentives exist (e.g., commercial organic fertilizers), the private costs paid by farmers to adopt such technologies for the production of high-quality agricultural products can be compensated by higher market transaction prices, while the expectation of obtaining higher private returns can further motivate farmers to adopt green technologies due to the existence of premium markets for high value-added and high-quality agricultural products [22]. However, farmers often do not receive a premium for high-quality agricultural products due to poor information traceability systems for such products and the difficulty of monitoring opportunistic behaviors in the production process, thereby resulting in a lack of incentives for adoption.

In this case, on the one hand, cooperatives can strengthen the mechanism of common property rights through dividends. Although dividends do not occur frequently, this does not prevent them from enhancing farmers’ membership identity, stabilizing the contractual relationship of transactions, or prompting farmers to follow incentive constraint norms to integrate and sell scattered products and achieve vertical integration upstream of the value chain. On the other hand, compared with smallholder farmers, cooperatives are more likely to connect with downstream value chain subjects, such as supermarkets, that are willing to pay high prices for high-quality agricultural products and establish a connection between farmers and premium markets for high-quality agricultural products, thus enhancing farmers’ private marginal returns and strengthening their economic incentives to adopt green technologies, thus shifting from passive adoption via compliance with binding norms to active adoption by seeking market returns.

As an example of green technologies without effective market incentives, water-saving irrigation technologies lack an effective irrigation water trading market, which makes it difficult for the scarcity value of irrigation water resources to be reflected in prices, while there is uncertainty regarding the realistic benefits of farmers’ adoption of such technologies, the economic incentives are insufficient, willingness to adopt is low, and the effect of water-saving incentive policies has been unsatisfactory [23,24]. Irrigation wells are important supports for farmers in adopting water-saving technologies. Publicly owned wells have public goods properties because they cover a large group of people and are prone to free-riding [25]. Therefore, incentives to invest in water-saving equipment are insufficient. Although privately-owned wells have clear property rights, investments in private wells not only require large amounts of capital but also face resource regulation policy constraints, such as well-drilling controls [26,27,28]. This is either unaffordable or unavailable to most smallholder farmers.

Conversely, cooperatives’ “acquaintance society” and peer-monitoring mechanism make individual water use behavior easy to observe and rights and responsibilities easy to define; further, cooperatives’ water stakeholders have mutual monitoring incentives, creating incentives and constraints similar to those in private property rights. Therefore, cooperative wells with shared ownership have greater incentives to adopt water-saving irrigation technologies.

Therefore, in this study, the use of commercial organic fertilizer technology was considered to represent green technologies for which there is an effective market incentive, and water-saving irrigation technologies to represent green technologies for which there is no effective market incentive. We discuss the effect of farmers’ participation in cooperatives on their adoption of these two types of green technologies, with “whether to adopt commercial organic fertilizer” and “whether to adopt water-saving irrigation technology” as outcome variables.

Table 1 shows that the adoption rates of commercial organic fertilizer and water-saving irrigation technologies were significantly higher among cooperative-member farmers than among non-member farmers. Therefore, this study’s findings have practical significance. The values and statistical descriptions of these two variables are shown in Table 2.

#### 2.3.2. Explanatory Variable

The core explanatory variable in this study was whether farmers participated in cooperatives. The literature has analyzed the effect of cooperative participation on farm households, usually using “cooperative membership” as an identifying variable [16]. In this paper, the household was the basic unit of the study rather than the individual farmer, and determine whether the household participates in a cooperative based on the household’s response to the question “Does your household participate in a cooperative?”

Moreover, given the recent emergence of “shell cooperatives” aiming to obtain government subsidies, this paper identifies “shell cooperatives” based on the household’s response to the question “Does your cooperative provide at least one production and business-related service to its members?” and attempt to eliminate the interference.

In addition, to comply with the principle of “cause before effect” in determining causality, this study tries to confirm the time of participation in the cooperatives. Thus, farmers who participated in a cooperative were asked “In which year did your family participate in the cooperative?” As the data in this paper reflect the production and operation information of farmers in 2017, farmers who participated in cooperatives before 2017 were included in this study.

In summary, the treatment group obtained in this paper comprised farmers who participated in non-shell cooperatives in 2017 and before, while the control group comprised farmers who did not participate in shell or non-shell cooperatives up to 2017. The core explanatory variable of this paper, “whether farmers participate in cooperatives,” is a dummy variable.

#### 2.3.3. Control Variables

As suggested by the previous literature, this study includes factors that may affect farmer households’ adoption of green technologies as control variables, which mainly include four aspects: personal characteristics of the household head, household characteristics, production and operation characteristics, and risk preference and risk perception. Table 2 presents the definitions and descriptive statistics of each variable.

Some studies show that the age and education of the household head, as the main decision-maker in the household, have important effects on household production and management behavior [29,30]. Differences in household characteristics, such as household support ratio, social capital, economic level, and part-time workers are also important causes of differences in farm households’ business behavior [31,32,33]. Production and management characteristics (e.g., the scale of cultivation, degree of land fragmentation, quality of cultivated land, land ownership, crop type, animal husbandry, and degree of commercialization) are closely related to farmers’ production behavior and can impact their technology adoption [34,35]. Further, agriculture has a high market and natural risk, while farmers’ risk preferences and risk perceptions (including natural risk perceptions and market risk perceptions) are important factors influencing their willingness to invest and risk management approaches, which can, in turn, affect their technology adoption behaviors [36].

Additionally, this study proposes the use of an endogenous transformation model for regression analysis; the identification variable is unique to the endogenous transformation model when dealing with the choice and outcome equations, while the identification variable affects the choice equation but not the outcome equation. In this study, the identification variable (similar to an instrument variable) was “whether a farmer has heard of cooperative law”. We believe that whether a farmer has heard of the cooperative law affects whether they join the cooperative but does not affect their green technology adoption behavior.

## 3. Empirical Results

### 3.1. Regression Results

Table 3 and Table 4 reports the estimation results of the OLS, Probit, and ESP models for the effect of farmers’ cooperative participation on their adoption of commercial organic fertilizers and water-saving irrigation technologies. The estimation results of the OLS and Probit models show that for the adoption of commercial organic fertilizers, the regression coefficients of the independent variables are 0.164 and 0.448, respectively, which are both statistically significant at the 1% levels, respectively. Comparing the regression results of the OLS and Probit models, it can be seen that the regression coefficients of the independent variables are positive and pass the significance test, but they differ significantly. For the adoption of water-saving irrigation, the regression coefficients of the OLS and Probit models were 0.062 and 0.367, respectively, with the former being significant at the 10% level and the latter not passing the significance test. This reflects the shortcomings of the OLS and Probit estimation methods in dealing with endogeneity.

The results of the ESP model showed that the residual correlation coefficients were all negative and significant at the 1% level; the joint independent likelihood ratios passed the significance tests at the 10% statistical levels, respectively, while the Wald test values were all significant at the 1% statistical level, thus rejecting the original hypothesis that the selection and outcome equations are independent of each other. Therefore, it is necessary to correct the sample selection bias caused by unobservable factors. The selection of the ESP model was appropriate.

The results of the OLS and Probit models show that participation in cooperatives has a significant positive effect on the adoption of commercial organic fertilizers by farmers, as well as a significant positive effect on soil fertility and market risk perception. At the same time, total household assets, land fragmentation, planting structure, degree of marketization, land title, and irrigation constraints had significant effects on the adoption of water-saving irrigation technologies. The results of the ESP model showed that the adoption of green technologies was influenced by a more complex set of variables.

Table 5 presents the estimated treatment effects of the ESP model on the adoption of commercial organic fertilizers and water-saving irrigation technologies. The average treatment effect (ATT) for the treatment group adopting commercial organic fertilizer was 0.332, the average treatment effect (ATU) for the control group was 0.625, and the average treatment effect (ATE) for all farmers was 0.474, all of which were statistically significant at the 1% level. This indicates that for the treatment group, the probability of applying commercial organic fertilizer was 33.2% higher if farmers participated in a cooperative, compared with a non-participation scenario. For the control group, the probability of applying commercial organic fertilizer was 62.5% higher if farmers participated in a cooperative. Further, the probability of applying commercial organic fertilizer was 47.4% higher if the overall sample participated in cooperatives. Notably, the average treatment effect of cooperative participation was higher in the control group than in the treatment group. It is noteworthy that the average treatment effect of participation in the cooperative was higher for the control group than for the treatment group. The possible reason for this is that for the treatment group, the very act of joining a cooperative implies that such farmers are more professional, more technologically literate, more inclined to adopt new technologies, and have the capacity to adopt new technologies on their own, even without joining a cooperative. For the control group, the act of not joining a cooperative may imply that such farmers are less technologically literate or competent and would not have access to new technologies if they had not joined a cooperative. This implies that after constructing the counterfactual framework, participating co-operatives significantly increased the probability of non-members adopting commercial organic fertilizer.

Meanwhile, the average treatment effect (ATT) for the treatment group adopting water-saving irrigation technologies was 0.185, the average treatment effect (ATU) for the control group was 0.192, and the average treatment effect (ATE) for all farmers was 0.189, all of which are statistically significant at the 1% level. This indicates that for the treatment group, the probability of adopting water-saving irrigation technology was 18.5% higher if farmers participated in a cooperative, compared with a non-participation scenario. For the control group, the probability of adopting water-saving irrigation technology was 19.2% higher if they participated in a cooperative. Additionally, the probability was 18.9% higher if the entire sample participated in a cooperative. Notably, the average treatment effect of cooperatives was higher in the control group than in the treatment group. The same story happens with the adoption of water-saving irrigation technologies. The possible reasons are similar. However, as irrigation facilities generally cover a larger group of users, the adoption of water-saving irrigation technologies is not as independent as the adoption of commercial organic fertilizer technologies, but rather group-based. Therefore, the impact of participating cooperatives on the adoption of water-saving irrigation technologies is relatively modest.

These results show that participating in cooperatives significantly promoted farmers’ adoption of green technologies for which effective market incentives exist (commercial organic fertilizer technologies, in this case) and those for which effective market incentives do not exist (water-saving irrigation technologies, in this case). These results are in general agreement with the findings of some of the existing research [15,16].

### 3.2. Robustness Test

This study uses a split-sample regression to test the robustness of the effects of farmers’ participating in cooperatives on their adoption of commercial organic fertilizers and water-saving irrigation technologies. Since differences in farmers’ resource endowments may affect their green technology adoption behavior, this study examined the average treatment effect for farmers with different planting sizes (see Table 6). As shown in Table 6, the direction and significance of the effects of ATT and ATE on the adoption of commercial organic fertilizer and water-saving irrigation technologies by farmers with different planting sizes were consistent. This indicates that cooperative participation significantly promoted the adoption of both commercial organic fertilizers and water-saving irrigation technologies by farmers at different planting scales, and the findings of this study are robust.

### 3.3. Analysis of Potential Mechanisms of Farmers’ Cooperative Participation Influence on Their Adoption of Green Technologies

The pathways through which cooperatives can influence farmers’ adoption of green technologies lie in the following areas. First, as mentioned above, for green technologies with effective market incentives, cooperatives can directly use optimal economic incentives to stimulate farmers’ willingness to adopt and reduce their tendency to adopt opportunistic behaviors by emphasizing the mechanism of shared property rights, thus increasing the probability of farmers’ adoption of green technologies. For example, in the case of commodity organic fertilizer technology, participating in cooperatives can effectively establish a connection between farmers and the premium market. Premium sales enhance farmers’ income from adopting commodity organic fertilizer and strengthen the economic incentive for them to adopt green technology, thus enhancing the level of farmers’ adoption of the technology. Simultaneously, cooperatives can strengthen the mechanism of common property rights through a dividend mechanism, enhance farmers’ awareness of property rights and membership identity, motivate farmers to actively comply with higher green technology specifications, reduce opportunistic behavior, and form positive incentives for members to adopt commercial organic fertilizers.

Second, for green agricultural technologies without effective market incentives (e.g., water-saving irrigation technologies), although there is no specific trading market or the development of such a market is seriously lagging, cooperatives can clarify farmers’ ownership of water-saving special assets and the corresponding right to use irrigation water through the common property rights mechanism, improve farmers’ access to public resources, and establish certain constraint mechanisms. Moreover, cooperatives can reduce farmers’ adoption costs through unified procurement of consumables and other socialized services, and create certain economic incentives for them to adopt key technologies. Additionally, cooperatives can establish information transfer mechanisms through training and technical services to improve farmers’ awareness of water conservation, their ability to adopt water-saving irrigation technologies, and their green technology literacy, thus promoting farmers’ adoption behavior.

## 4. Conclusions

This study analyzed the effect of cooperatives in promoting farmers’ adoption of green technologies using an ESP model based on micro-survey data from farmers in Shandong, Hebei, Sichuan, and Gansu provinces in 2018, while also conducting robustness tests and impact mechanism analysis. Based on the results of the empirical analysis, the following conclusions were drawn.

Participation in cooperatives helps promote farmers’ adoption of green technologies, regardless of whether there is an effective market incentive to adopt such technologies. For green technologies with effective market incentives, participation in cooperatives helps strengthen economic incentives for farmers to adopt such technologies by establishing links between farmers and the premium market, as well as dividend mechanisms that enhance farmers’ awareness of property rights and membership identity. For green technologies that do not have effective market incentives, cooperatives can still clarify farmers’ ownership of dedicated assets and the right to use corresponding resources through common property rights and reduce farmers’ green technology adoption costs through socialized services such as unified procurement of consumables. Additionally, cooperatives can improve farmers’ abilities and knowledge to adopt green technology by providing them with training and technical services, thus promoting their adoption of green technology.

To make cooperatives effective tools for promoting farmers’ adoption of green technologies, policymakers should focus on the following three aspects. First, policies promoting green technology and supporting farmers’ cooperatives should be promoted in conjunction to provide institutional support for cooperatives to implement multiple mechanisms to promote green agricultural development. A clear and effective incentive and penalty system should be established, focusing on the standardization of cooperatives, enhancing the benefit-sharing mechanism between cooperatives and ordinary members, and using market mechanisms to guide cooperatives to actively establish economic incentives to promote farmers’ adoption of green technologies; this way, cooperatives could facilitate the effective connection between farmers and the market to transform the value of green technology applications into transaction benefits.

Second, it is necessary to actively guide cooperatives to innovate the property rights system of special assets related to green technologies and promote the adoption of green technologies by taking advantage of common property rights. Further, it is paramount to accelerate and improve the confirmation and registration of common property rights of assets dedicated to green technologies, as well as the establishment of a trading market to transform the application value of green technologies into real benefits to strengthen the economic incentive for farmers to participate in the governance of public resources and the adoption of green technologies.

Third, importance should be attached to cooperatives as promotors of green technologies, while enhancing their members’ green technology literacy and cooperatives’ social norm guidance. By using cooperatives’ localized information advantages, green technologies can be actively promoted and adjusted to farmers’ actual local conditions. Cooperatives can guide farmers in adjusting their understanding of relevant policies and expectations of the results of green technology adoption, which could promote the achievement of green agricultural development by strengthening farmers’ subjective awareness of the adoption of such technologies and enhancing their cognitive awareness and ability to use advanced green agricultural technologies.

The paper has two major limitations. First, the data used in the paper come from a survey of 411 farmers in four Chinese provinces. The results may not be generalizable to other regions. Second, the relationship between farmers’ cooperative participation and the adoption of various green technologies may be more diverse and complicated. The costs of adopting a particular green technology may influence farmers’ adoption. Furthermore, the farmers may adopt multiple green technologies, and the different green technology adoptions may affect each other. However, due to data limitations, we are not able to do a more comprehensive analysis. In future studies, the multidimensional mechanisms and key links of farmers’ cooperative participation to promote their adoption of multiple green technologies can be further considered and incorporated into the research framework.

## Figures and Tables

**Table 1:** Comparative analysis of green technology adoption behaviors among cooperative-member farmers and non-member farmers.

Adoption of	Overall	Cooperative Members			Non-Members		*t*-Test
	N	%	N	%	N	%	Difference
commercial organic fertilizers	199	48.42	130	62.20	69	34.16	28.04 ***
water-saving irrigation	81	19.71	63	30.14	18	8.91	21.23 ***

Note: *** indicates significance at the 1% level.

**Table 2:** Descriptive statistics of the main variables.

Variable Type	Variables	All	Member Group	Non-Member Group	*p*-Value
		N = 411	N = 209	N = 202	
Outcome variables	Adoption of commercial organic fertilizers	48.42	62.20	34.16	<0.001
	Adoption of water-saving irrigation technologies	19.71	30.14	8.91	<0.001
Control variables					
Personal Characteristics	Age (years)	52.68	52.58	52.78	0.571
	Education level				0.023
	*Elementary school or below*	37.71	33.01	42.57	
	*Junior High School*	45.99	48.80	43.07	
	*High School*	15.33	16.75	13.86	
	*College and above*	0.97	1.44	0.50	
	Gender (male)	0.97	0.97	0.96	0.27
Family Characteristics	Number of visits to friends and relatives during the Spring Festival	43.20	46.66	39.63	0.081
	Household dependency ratio (dependent population/total household size)	0.70	0.70	0.69	0.417
	Total household assets = ln (valuation of house and land value + valuation of agricultural facilities + financial assets)	12.38	12.64	12.11	<0.001
Production and management characteristics	part-time business	65.45	62.2	68.81	0.16
	Planting area (mu)	6.90	7.79	5.98	0.002
	Degree of land fractionation (average acres per parcel)	2.57	2.96	2.16	0.007
	The main crops are fruits and vegetables	35.28	51.2	18.81	<0.001
	Land Quality				0.065
	*Good land*	61.07	65.07	56.93	
	*General land*	33.82	31.10	36.63	
	*Poor land*	5.11	3.83	6.44	
	Proportion of agricultural products produced for sale (%)	91.54	94.40	88.77	0.002
	Animal husbandry	0.17	0.22	0.12	0.003
	Land Title				<0.001
	*all owned land*	62.04	75.60	48.02	
	*owned land + leased land*	30.17	18.66	42.08	
	*all leased land*	7.79	2.39	13.37	
	Irrigation condition constraints				0.016
	*irrigation is easier than others*	28.22	33.01	23.27	
	*medium difficulty of irrigation*	65.69	61.72	69.80	
	*irrigation is more difficult than others*	6.08	5.26	6.93	
	Risk attitude				0.024
	*risk aversion*	54.74	50.72	58.91	
	*risk-neutral*	7.30	6.22	8.42	
	*risk appetite*	37.96	43.06	32.67	
	Subject to credit constraints	38.20	39.71	36.63	0.26
	Concerned about losses due to natural risks	60.58	58.37	62.87	0.176
	Concerned about losses due to market risk	75.67	74.64	76.73	0.311
	Province				<0.001
	*Sichuan*	19.95	29.19	10.40	
	*Shandong*	28.71	41.63	15.35	
	*Hebei*	18.98	5.74	32.67	
	*Gansu*	32.36	23.44	41.58	
Identification Variable	The head of the household has heard of the cooperative law	37.47	55.98	18.32	<0.001

**Table 3:** OLS, Probit, and ESP estimates of farmers’ adoption of commercial organic fertilizer technology vis-à-vis their cooperative membership.

	OLS	Probit	ESP		
			Select Equation	Processing Group	Control Group
Cooperative members	0.164 ***	0.448 ***	-	-	-
	(0.056)	(0.157)	-	-	-
Age	−0.003	−0.008	−0.013 *	−0.013	−0.013
	(0.002)	(0.007)	(0.007)	(0.009)	(0.010)
Education level	-	-	−0.051	0.028	−0.069
	-	-	(0.111)	(0.125)	(0.142)
*Junior High School*	0.055	0.156	-	-	-
	(0.057)	(0.165)	-	-	-
*High School*	0.053	0.131	-	-	-
	(0.075)	(0.215)	-	-	-
*College and above*	0.216	0.614	-	-	-
	(0.247)	(0.737)	-	-	-
Male	−0.151	−0.445	0.413	0.382	−1.600 ***
	(0.130)	(0.361)	(0.376)	(0.402)	(0.553)
Credit constraints	−0.063	−0.176	0.095	−0.624 ***	0.496 **
	(0.052)	(0.149)	(0.159)	(0.175)	(0.235)
Social networks	0.000	0.000	−0.001	−0.000	0.000
	(0.000)	(0.001)	(0.001)	(0.001)	(0.002)
Family support ratio	0.047	0.138	0.374	−0.188	0.107
	(0.100)	(0.290)	(0.314)	(0.330)	(0.423)
Total household assets	0.009	0.037	0.120 *	0.055	−0.052
	(0.023)	(0.068)	(0.073)	(0.079)	(0.103)
Part-time business	0.012	0.028	0.142	−0.017	−0.333
	(0.056)	(0.158)	(0.166)	(0.178)	(0.236)
Planting area	0.005	0.013	0.049 ***	0.004	0.007
	(0.005)	(0.014)	(0.018)	(0.016)	(0.029)
Land fractionation	−0.004	−0.010	−0.056 *	0.004	−0.046
	(0.010)	(0.029)	(0.030)	(0.030)	(0.061)
Planting structure	0.068	0.183	0.661 ***	−0.222	0.142
	(0.071)	(0.201)	(0.201)	(0.219)	(0.347)
Soil fertility	0.079 *	0.237 **	−0.112	0.338 **	0.012
	(0.041)	(0.118)	(0.122)	(0.135)	(0.167)
Degree of marketization	0.000	0.000	−0.008	0.004	0.023 **
	(0.002)	(0.005)	(0.005)	(0.005)	(0.010)
Animal husbandry	−0.001	0.004	0.113	−0.345	0.004
	(0.082)	(0.238)	(0.232)	(0.248)	(0.373)
Land title	−0.050	−0.139	0.445 ***	−0.210	−0.239
	(0.044)	(0.128)	(0.136)	(0.134)	(0.254)
Risk attitude	0.036	0.104	0.125 *	0.038	−0.013
	(0.026)	(0.074)	(0.076)	(0.086)	(0.116)
Natural risk perception	−0.016	−0.045	0.149	0.031	−0.166
	(0.051)	(0.146)	(0.157)	(0.179)	(0.209)
Market risk perception	0.150 ***	0.432 ***	−0.018	0.500 ***	0.075
	(0.057)	(0.166)	(0.171)	(0.181)	(0.251)
Province	Controlled	Controlled	Controlled	Controlled	Controlled
Heard of the cooperative law			0.943 ***		
			(0.144)		
Constant term	0.321	−0.652	−1.252	−0.176	1.700
	(0.418)	(1.186)	(1.297)	(1.370)	(1.922)
Pseudo R2	0.137	0.147			
ρ1	-	-	-	−1.000 *** (0.000)	
ρ0	-	-	-	−0.236 (0.376)	
Maximum likelihood estimate	-	-	-	−425.154	
Wald test value	-	-	-	137.61 ***	
Joint independent likelihood ratio test	-	-	-	5.910 *	
Number of observations	411	411	411	209	202

Note: *, **, and *** indicate significance at the 10%, 5%, and 1% levels, respectively.

**Table 4:** OLS, Probit, and ESP estimates of farmers’ adoption of water-saving irrigation technologies vis-à-vis their cooperative membership.

	OLS	Probit	ESP		
			Select Equation	Processing Group	Control Group
Cooperative members	0.062 *	0.367	-	-	-
	−0.036	−0.247	-	-	-
Age	0.000	0.007	−0.008	0.005	0.036
	−0.002	−0.011	−0.008	−0.013	−0.035
Education level	0.021	0.25	0.013	0.153	0.121
	−0.023	−0.172	−0.114	−0.204	−0.498
Credit constraints	−0.037	−0.239	0.216	0.063	−0.482
	−0.034	−0.249	−0.168	−0.277	−0.961
Social networks	−0.000 *	−0.003	−0.001	−0.000	−0.005
	−0.001	−0.002	−0.001	−0.002	−0.006
Family support ratio	−0.068	−0.544	−0.273	−0.189	−1.404 *
	−0.066	−0.443	−0.333	−0.539	−0.769
Total household assets	0.055 ***	0.489 ***	0.105	0.407 **	0.732 *
	−0.015	−0.15	−0.072	−0.198	−0.43
Part-time business	−0.08 **	−0.403 *	0.12	−0.324	−1.282 **
	−0.036	−0.235	−0.18	−0.275	−0.589
Planting area	−0.004	−0.029	0.827 **	0.298	1.185
	−0.003	−0.026	−0.21	−0.456	−0.827
Land fragmentation	0.024 ***	0.107 **	−0.055	0.191 ***	−0.137
	(0.006	−0.05	−0.034	−0.073	−0.202
Planting structure	0.173 ***	0.697 ***	0.048 ***	−0.0650 *	0.088
	−0.044	−0.262	−0.017	−0.034	−0.073
Soil fertility	−0.009	−0.01	−0.073	0.012	−0.979
	−0.027	−0.215	−0.13	−0.27	−0.665
Degree of marketization	0.004 ***	0.059 **	−0.008	0.047 **	0.510 **
	(0.001	−0.024	−0.005	−0.024	−0.216
Land title	0.070 **	0.339*	0.427 ***	0.069	0.314
	−0.029	−0.178	−0.139	−0.229	−0.767
Risk attitude	0.027	0.101	0.12	0.066	−0.324
	−0.017	−0.11	−0.081	−0.134	−0.263
Irrigation constraints	−0.074 **	−0.487 **	0.028	−0.395	−1.186 *
	−0.032	−0.207	−0.155	−0.251	−0.644
Natural risk perception	0.012	0.133	-	-	-
	−0.033	−0.227	-	-	-
Market risk perception	−0.019	−0.227	-	-	-
	(0.038	−0.269	-	-	-
Province dummies	Controlled	Controlled	Controlled	Controlled	Controlled
Heard of the cooperative law	-	-	0.999 ***	-	-
	-	-	−0.165	-	-
Constant term	−0.748 ***	−12.41 ***	−1.625	−13.32	−56.03 **
	−0.277	−3.297	−1.334	−124.9	−23.98
Pseudo R2	0.432	0.517	-	-	-
LR chi2	-	210.81	-	-	-
ρ1	-	-	-	−0.590 *** (0.501)	
ρ0	-	-	-	−0.999 *** (0.000)	
Maximum likelihood estimate	-	-	-	−270.506	
Wald test value	-	-	-	140.710 ***	
Joint independent likelihood ratio test	-	-	-	7.950 **	
Observations	411	411	411	209	202

Note: *, **, and *** represent 10%, 5%, and 1% significance levels, respectively, with standard errors in parentheses.

**Table 5:** Estimated treatment effects of farmers’ cooperative participation on their adoption of commercial organic fertilizers and water-saving irrigation technologies (ESP estimation results).

Variable Name	ATT (N = 209)	ATU (N = 202)	ATE (N = 411)
Adoption of commercial organic fertilizers	0.332 *** (0.014)	0.625 *** (0.012)	0.474 *** (0.017)
Adoption of water-saving irrigation technologies	0.185 *** (0.021)	0.192 *** (0.018)	0.189 *** (0.013)

Note: *** represents 1% significance level, with standard errors in parentheses.

**Table 6:** Analysis of planting scale differences in the average treatment effect of commercial organic fertilizers.

Adoption of	Planting Scale	N	ATE	SE	N	ATT	SE
commercial organic fertilizers	<10 mu	325	0.482 ***	0.014	156	0.312 ***	0.026
	10 mu~50 mu	86	0.444 ***	0.030	53	0.391 ***	0.045
water-saving irrigation technologies	<10 mu	325	0.208 ***	0.013	156	0.207 ***	0.022
	10 mu~50 mu	86	0.114 ***	0.036	53	0.119 ***	0.053

Note: *** represents 1% significance level.

## Data Availability

Not applicable.
